# Assessment of a targeted resequencing assay as a support tool in the diagnosis of lysosomal storage disorders

**DOI:** 10.1186/1750-1172-9-59

**Published:** 2014-04-25

**Authors:** Ana Fernández-Marmiesse, Marcos Morey, Merce Pineda, Jesús Eiris, Maria Luz Couce, Manuel Castro-Gago, Jose Maria Fraga, Lucia Lacerda, Sofia Gouveia, Maria Socorro Pérez-Poyato, Judith Armstrong, Daisy Castiñeiras, Jose A Cocho

**Affiliations:** 1 Unidad Diagnóstico y Tratamiento de Errores Congénitos del Metabolismo (Servicio de Neonatología), Facultad de Medicina y Odontología de la Universidad de Santiago de Compostela, 15706 Santiago de Compostela, La Coruña, Spain; 2 Neuropediatra Fundación Hospital San Juan de Dios, CIBERER, Barcelona, Spain; 3 Servicio de Neuropediatría, Hospital Clínico Universitario de Santiago de Compostela, Facultad de Medicina y Odontología de la Universidad de Santiago de Compostela, Santiago de Compostela, La Coruña, Spain; 4 Unidade de Bioquímica Genética, Centro de Genética Médica Jacinto Magalhães, Centro Hospitalar do Porto, Porto, Portugal; 5 Unidad de Neuropediatría. Hospital Clínico Universitario Marqués de Valdecilla, Santander, Spain; 6 Servicio de Genética Molecular, Hospital San Juan de Dios, Barcelona, Spain

**Keywords:** In-solution enrichment, Targeted resequencing, Lysosomal storage disorders, Diagnostic odysseys

## Abstract

**Background:**

With over 50 different disorders and a combined incidence of up to 1/3000 births, lysosomal storage diseases (LSDs) constitute a major public health problem and place an enormous burden on affected individuals and their families. Many factors make LSD diagnosis difficult, including phenotype and penetrance variability, shared signs and symptoms, and problems inherent to biochemical diagnosis. Developing a powerful diagnostic tool could mitigate the protracted diagnostic process for these families, lead to better outcomes for current and proposed therapies, and provide the basis for more appropriate genetic counseling.

**Methods:**

We have designed a targeted resequencing assay for the simultaneous testing of 57 lysosomal genes, using in-solution capture as the enrichment method and two different sequencing platforms. A total of 84 patients with high to moderate-or low suspicion index for LSD were enrolled in different centers in Spain and Portugal, including 18 positive controls.

**Results:**

We correctly diagnosed 18 positive blinded controls, provided genetic diagnosis to 25 potential LSD patients, and ended with 18 diagnostic odysseys.

**Conclusion:**

We report the assessment of a next–generation-sequencing-based approach as an accessory tool in the diagnosis of LSDs, a group of disorders which have overlapping clinical profiles and genetic heterogeneity. We have also identified and quantified the strengths and limitations of next generation sequencing (NGS) technology applied to diagnosis.

## Introduction

Lysosomal storage disorders (LSDs) are rare diseases with a combined incidence of ~1 in 1500 to 7000 live births [1,2]. This group of inborn errors of metabolism encompasses >50 different diseases, each characterized by the accumulation of specific substrates [3-6]. Generally, newborns with LSDs appear normal at birth and symptoms develop progressively over the first few months of life. Late-onset juvenile and adult forms of LSDs, resulting from chronic substrate accumulation, also occur, but due to their varied signs and symptoms can have a delayed diagnosis. The recent development and availability of enzyme-replacement therapy (ERT) for several LSDs means that diagnosis early in the clinical process is of particular relevance [7]. Most importantly, early genetic diagnosis can provide parents with realistic information about their child’s prognosis, enable appropriate genetic counseling about future pregnancies, and prevent ‘diagnostic odysseys’ for families [8].

Because the clinical features of many LSDs overlap, establishing a diagnosis solely on the basis of clinical presentation is difficult. Until recently, clinicians have had different ways of approaching LSD diagnosis. The first option has been laboratory assays based on detection of the storage product. Although many of the clinical symptoms of different LSDs result primarily from substrate storage anomalies, presentation varies widely, depending on type, quantity, location, and time of extraction of the accumulated storage material, thus frequently giving rise to false negatives in biopsy analysis. Tests for elevated levels of secreted substrate material are routinely used to examine the pattern of glycosaminoglycans and oligosaccharides in patients suspected of having mucopolysaccharidoses (MPS) or disorders that present with oligosacchariduria. Although urine screens are very sensitive, affected individuals with normal urine screens have been reported mainly in young and adults; thus, when there is a strong index of suspicion, normal urine screening results should still be followed by enzyme analysis [9-11]. Enzyme activity detected in blood spots, either individually or simultaneously, is useful in the diagnosis of a small number of LSDs, but needs verification with a second type of assay; while measurement of enzyme activity in leukocytes and plasma serves this purpose for most LSDs, a proportion of cases may also not be detected using this method. Other limitations with enzyme activity tests is that they cannot detect heterozygous carriers of a disease, and are not suitable for potentially oligogenic LSDs (not described so far for LSD but recognized for retinitis pigmentosa, deaffness or ciliopathies) [12]. All of the above methods are laborious, time-consuming, and require accurate clinical diagnosis to reduce the number of enzymatic assays used for each patient. Moreover, all these techniques are semi-quantitative and subject to high variability, leading to false positives and negatives.

In summary, diagnosis of LSDs represents a challenge for clinicians and can take several years. Even reaching a diagnosis with traditional techniques, a genetic diagnosis also has to be made in order to provide the family with appropriate genetic counseling, itself arguably as important as the diagnosis. Genetic analysis is usually not performed as the primary screening tool in the diagnosis of LSD due to the cost and delay incurred by the sequential genetic tests necessary to diagnose any particular disorders. However, with the availability of next-generation sequencing (NGS) technologies, a genetically based diagnosis can be completed in 4 or 6 weeks, while reducing the cost to that of Sanger sequencing a single gene [13-15]. Here, we present the results of a pilot project to evaluate the application of NGS to mutation screening in a diagnostic context. We show the strengths and limitations of this approach and, although this assay would never suffice as the sole diagnostic tool, we propose it as a useful adjunct to diagnosis for specialists in everyday clinical management who might suspect an LSD, given its ability to provide accurate information in a short time.

## Methods

### Ethics statement

The study protocol adhered to the tenets of the Declaration of Helsinki and was approved by the local Ethics Committee (Comité Ético de Investigación Clínica de Galicia - CEIC). Informed written consent was obtained from each study participant. Index patients underwent a full neurological examination in each source hospital. Whenever available, blood samples from affected and unaffected family members were collected for co-segregation analysis.

### Probands

A total of 84 probands were collected from different institutions in Spain and Portugal, including 18 positive controls, and 66 patients with a suspected LSD. Positive controls underwent biochemical test and Sanger sequencing. Analyses of 13 controls and 33 patients were performed with SOLiD and 5 controls and 33 patients with Illumina platform.

### LSD diagnostic suspicion index

Subjective parameter chosen by the clinical specialists who managed each case. We asked them to choose between three degree of suspicion: high (you believe your patient has a lysosomal disease with a high probability due to biochemical or clinical data), low (your main suspicion is another condition but there is a low probability that even if it is a lysosomal disease and it is important to discard it), medium (in the differential diagnosis are lysosomal diseases).

### Capture array design, library construction, and NGS

A custom Sure Select oligonucleotide probe library was designed to capture the 551 exons and exon-intron-boundaries of 57 genes known to be associated with LSDs, according to GeneReviews (NCBI) (Table 1) [16]. Design includes all transcripts from each target gene. The eArray web-based probe design tool was used for this purpose [17]. The following parameters were chosen for probe design: 120 bp length for baits, 5X probe-tiling frequency, and 20 bp overlap in restricted regions identified by the implementation of eArray’s Repeat Masker program. A total of 5037 unique baits, covering 183,440 bp, were generated and synthesized by Agilent Technologies (Santa Clara, CA, USA). Sequence capture, enrichment, and elution were performed according to the manufacturer’s instructions.

**Table 1:** Genes included in the NGS-LSD assay and their associated disorders

**Gene**	**Lysosomal storage dirorder**
SMPD1	Niemann-Pick Disease, Type A and B
NPC1	Niemann-Pick Disease, Type C1
NPC2	Niemann-Pick Disease, Type C2
LIPA	Wolman Disease
GLA	Fabry Disease
GLB1	GM1-Gangliosidosis, Type I, II, III
GM2A	GM2-Gangliosidosis, AB Variant
HEXA	Tay-Sachs Disease
HEXB	Sandhoff Disease
GBA	Gaucher Disease
GAA	Pompe Disease
IDUA	MPS I: Hurler/Scheie
IDS	MPS II: Hunter Syndrome
SGSH	MPS IIIA: Sanfilippo Type A
NAGLU	MPS IIIB: Sanfilippo Type B
HGSNAT	MPS IIIC: Sanfilippo Type C
GNS	MPS IIID: Sanfilippo Type D
GALNS	MPS IVA: Morquio A
GLB1	MPS IVB: Morquio B
ARSB	MPS VI: Maroteaux-Lamy Syndrome
GUSB	MPS VII: Sly Syndrome
HYAL1	MPS IX
ASAH1	Farber Disease
ARSA	Metachromatic Leukodystrophy
GALC	Krabbe Disease
PSAP	Prosaposin deficiency
NEU1	Mucolipidosis I: Sialidosis
FUCA1	Fucosidosis
LAMP2	Danon Disease: Glycogen Storage Disease IIB
LAMP3	Candidate Gene For LSD
GNPTAB	Mucolipidosis II Alpha/Beta
GNPTG	Mucolipidosis III Gamma
MCOLN1	Mucolipidosis IV: Sialolipidosis
MAN2B1	Mannosidosis, Alpha B, Lysosomal
MANBA	Mannosidosis, Beta A, Lysosomal
PPT1	Ceroid Lipofuscinosis, Neuronal, 1
TPP1	Ceroid Lipofuscinosis, Neuronal, 2
CLN3	Ceroid Lipofuscinosis, Neuronal, 3 (Batten Disease)
CLN5	Ceroid Lipofuscinosis, Neuronal, 5
CLN6	Ceroid Lipofuscinosis, Neuronal, 6
CLN7	Ceroid Lipofuscinosis, Neuronal, 7
CLN8	Ceroid Lipofuscinosis, Neuronal, 8
CLN10	Ceroid Lipofuscinosis, Neuronal, 10
CTSA	Galactosialidosis
CTNS	Cystinosis
SLC17A5	Sialic Acid Storage Disease
CTSK	Pyknodysostosis
NAGA	Schindler Disease
SUMF1	Multiple Sulfatase Deficiency
HPS1	Hermansky-Pudlak Syndrome Type 1
AP3B1	Hermansky-Pudlak Syndrome Type 2
HPS3	Hermansky-Pudlak Syndrome Type 3
HPS4	Hermansky-Pudlak Syndrome Type 4
HPS5	Hermansky-Pudlak Syndrome Type 5
HPS6	Hermansky-Pudlak Syndrome Type 6
DTNBP1	Hermansky-Pudlak Syndrome Type 7
BLOC1S3	Hermansky-Pudlak Syndrome Type 8

### SOLiD4 platform

Briefly, 3–4 μg of each genomic DNA was fragmented by sonication (Covaris S2, Massachusetts, USA), purified to yield 150–180 bp fragments and end-repaired. Adaptor oligonucleotides from Agilent technologies were ligated on repaired DNA fragments, which were then purified, size-selected by gel electrophoresis, nick-translated, and amplified by 12 PCR cycles. The libraries (500 ng) were then hybridized to the Sure Select biotinylated-RNA capture library for 24 h. After hybridization, washing, and elution, the captured fraction underwent 12 cycles of PCR amplification with barcoded primers followed by purification and quantification by qPCR. Forty-eight barcoded samples were then pooled in groups for sequencing on a SOLiD4 platform as single end 50 bp reads. 12 sample libraries were loaded per octet of SOLiD4 slide.

### HiSeq2000 platform

The library preparation for capturing of selected DNA regions was performed according to the SureSelect XT Target Enrichment System protocol for Illumina paired-end sequencing (Agilent). In brief, 3 μg of genomic DNA was sheared on a Covaris™ E220 focused-ultrasonicator. Fragment size (150-200 bp) and quantity were confirmed with an Agilent 2100 Bioanalyzer 7500 chip. The fragmented DNA was end-repaired, adenylated and ligated to Agilent indexing-specific paired-end adaptors. The DNA with adaptor-modified ends was PCR amplified (6 cycles, Herculase II fusion DNA polymerase) with SureSelect primers, quality controlled using the DNA 7500 assay specific for a library size of 250–350 bp, and hybridized for 24 hr at 65°C. The hybridization mixture was washed in the presence of magnetic beads (Dynabeads MyOne Streptavidin T1, Life Technologies), and the eluate PCR amplified (16 cycles) to add index tags using SureSelectXT Indexes for Illumina. The final library size and concentration was determined using an Agilent 2100 Bioanalyzer 7500 chip and sequenced on an Illumina HiSeq 2000 platform with a paired-end run of 2 × 76 bp, following the manufacturer’s protocol. 36 sample libraries were loaded in three lanes of HiSeq 2000.

### Data filtering and analysis pipeline

#### SOLiD4 platform

Image analysis and base calling was performed using the SETS (SOLiD experimental Tracking Software) pipeline to generate primary data. Sequence reads were aligned to the reference human genome UCSC hg19 using Life Technologies’ BioScope suite v1.3.1. Default parameters, recommended for targeted resequencing, were used. Variant calling was performed using two software programs in parallel: the diBayes alignment algorithm embeded in the Bioscope suite [18] and the Genome Analysis Toolkit (GATK) v1.5, a software package developed at the Broad Institute (Cambridge, MA) to analyze next-generation resequencing data [19]. The GATK Unified Genotyper is a state-of-the-art variant caller for NGS data and used extensively in human sequencing projects. The variant detection pipeline uses well-established statistical models for recalibration of the base quality score and variant calling. Low stringency parameters were selected to avoid false negatives although a high rate of false positives was expected.

#### HiSeq2000 platform

Base calling and quality control were performed on the Illumina Real Time Analysis (RTA) sequence analysis pipeline. Sequence reads were trimmed to keep only those bases with a quality index > 10 and then mapped to Human Genome build hg19 (GRCh37), using a Genome Multitool (GEM) [20] and allowing up to 4 mismatches. Reads not mapped by GEM were submitted to a last round of mapping with BLAT-like Fast Accurate Search Tool (BFAST) [21]. Uniquely mapping non-duplicate read pairs were locally realigned with GATK. Samtools suite [22] was used to call single nucleotide variants (SNVs) and short INDELs, taking into account all reads per position. Variants on regions with low mappability or variants in which there was not at least one sample with read depth ≥10 were filtered out.

### Sanger sequencing

To verify the DNA sequence variants detected by NGS, we amplified the target sites and flanking sequences of each variant with specific primers designed using the free software Primer3 v.0.4.0 [23]. Next, we sequenced the PCR products using the Sanger method to ascertain the precision of the variants identified by NGS. Sequencing reactions consisted of 1.0 μl of previously purified PCR products (ExoSAP-IT, USB, Cleveland, OH), 1 μl of each primer, and 1 μl of Big Dye Terminator v3.1 from the Cycle Sequencing kit (Applied Biosystems, Foster City, CA). The reactions were run in an ABI 3730 DNA Analyzer (Applied Biosystems, Foster City, CA). Analysis was performed with the Staden package free software.

### Detection of gross indels

A simple homemade Excel table was designed for detection of gross deletions and duplications encompassing one or more exons. The table contained coverage peak areas for each exon and patient. Ratios of successive peak areas between different patients were compared to identify homozygous and heterozygous gross indels. To establish the extremes of deletion it is necessary to perform cDNA Sanger sequencing studies.

### Filtering of annotated sequence data

We received the bam files and the annotated sequencing variants in Excel tables from the two platforms. Raw data were filtered with custom designed scripts using the free, open source statistical software R package [24]. We have submitted all novel variants included in the article to ClinVar database [25] from NCBI and to the Locus Specific Mutation Databases (LSMDs) from Human Genome Variation Society [26].

### Assessment of the pathogenicity of variants

The following criteria were applied to evaluate the pathogenic nature of novel variations identified by NGS: 1) stopgain, frameshift and splicing variants, considered the most likely to cause disease; 2) the presence of a second mutant allele, taken to indicate recessive inheritance; 3) cosegregation; 4) the absence of the variant in other samples; 5) frequency <0.01 in the 1000 g2012 database; and 6) for missense mutations, amino acid conservation and prediction of pathogenicity. To evaluate criterion 6), we used the freely available bioinformatics web tools: SIFT (Sorting Intolerant From Tolerant) [27], Polyphen-2 [28] and Mutation Taster [29]. To evaluate the possible effect of synonymous variant in gene splicing we used the Human Splicer Finding web tool [30].

## Results and discussion

### Statistical data from two platforms

Total target sequence length, including coding exons and exon-intron junctions from 57 LSD genes, was 183,440 bp. Coverage distribution across the designed region was replicated among the samples for each platform. The overall sequence depth was 456X for SOLiD4 and 7,416X for HiSeq2000. SOLiD4 yielded a total of up to 158,863,367 raw 50-mer reads with 87.28% mapped reads overall. A total of 94.5% of bases were covered by at least 20 reads and the mean percentage of bases not covered per sample was 2.6%. The HiSeq2000 run resulted in (1292,371,000) QC-passed reads with 88.47% mapped reads overall. A total of 99.97% of bases were covered by at least 20 reads, involving a percentage of target bases with less than 20 reads of only 0.03%.

### Limitations of the enrichment method

The most important challenge for this kind of enrichment is that local sequence architecture has a strong effect on the efficiency of DNA enrichment for individual exons [31]. Exons close to repetitive regions are not fully covered, because the eArray web tool does not design baits in repetitive regions. In our assay 5 exons were affected by this initial lack of baits. We tried to minimize this pitfall by using the 5X bait-tailing option, to reduce the gaps as much as possible. However, our results showed that such an approach overcame this problem only if coverage was increased maximally. Another significant limitation of the enrichment method is that coverage decreases dramatically, even to zero, for exons located in CpG islands. With 456X coverage, 22 of 57 genes showed gaps in coverage (<20X) in one exon, and 6 in more than one exon, with the most damaged genes being *IDUA*, *GBA*, and *GAA*. Thus, our NGS-LSD assay did not detect mutations in the *IDUA* gene in a patient with biochemically confirmed Hurler disease. With 7416X coverage no gaps were found in any of LSD genes. Therefore, to establish this assay as a diagnostic screening tool for routine use it will be essential to eliminate sequence gaps. Because enrichment failures with hybrid capture were reproducible, they may be amenable to rescue by individual PCRs or probe redesign.

### Filtering of raw data

The main challenge of using NGS for diagnostic applications will be in interpreting the massive number of genomic variants detected by sequencing platforms. Fast and reliable identification of causative variants will be crucial to the implementation of this technology in diagnostics. In our study, simple filters based on variant function and frequency, and careful selection of index cases versus controls, was found to be a useful way of discriminating between pathogenic mutations and background polymorphisms. Priority was given to variants considered to be specific to individual patients, based on the assumption that a mutation underlying such a monogenic disorder is highly penetrant and rare. It was also assumed that these mutations are likely to be coding and to have a major phenotypic effect.

**Figure 1:**
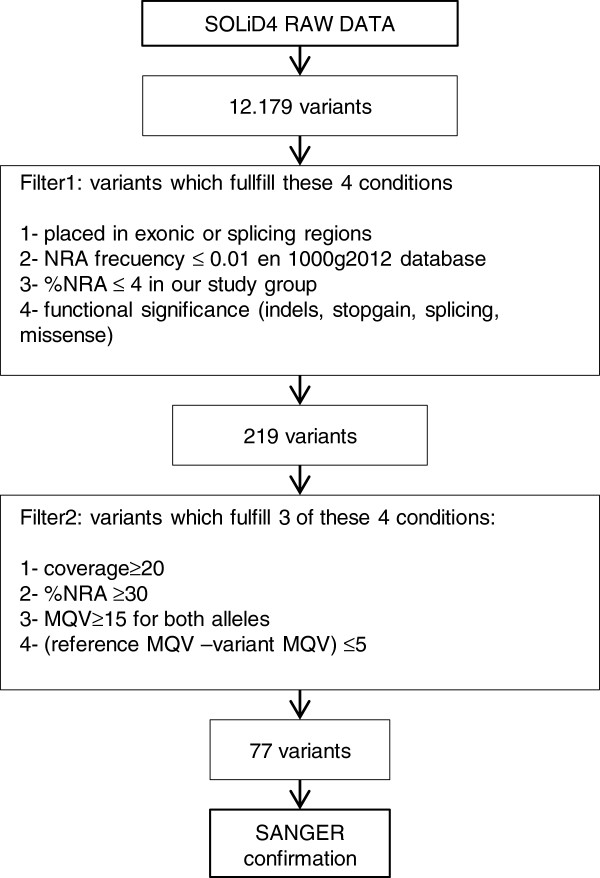
**Global flowchart of the filtering pipeline used for selection of most likely pathogenic mutations, starting from SOLiD4 raw data.** (NRA: non-reference allele).

From the 12,179 and 52,303 variants detected by the SOLiD4 and HiSeq platforms, respectively, successive filters were applied to reduce these numbers to 77 and 68. SOLiD4 raw data provided by Bioscope SNP calling software were filtered twice, using specifically designed R scripts (Figure 1). Filter 1 selected variants with the following specific features: 1) non-reference allele (NRA) frequency ≤0.01 in the 1000 g2012 database; 2) location in exonic or splicing regions; 3) non-synonymous, indels, stopgain, or splicing; and 4) NRA frequency ≤4% in our study group. This filter reduced the initial number of variants to 219. Filter 2 then discriminated between real and false-positive changes, by fulfilling at least 3 of following conditions: 1) coverage of position ≥20; 2) percentage of NRA reads ≥30; 3) mean quality values (MQV) of reference and variant ≥15; 4) [reference-variant] MQV ≤5. The number of variants was reduced to 77, implying a false-positive rate of 65%. Careful observation of the false-positive variants showed that they appeared in characteristic areas which either had a low coverage or a sudden drop in coverage, or were considered coverage ‘valleys’. Therefore such false positives were the result of coverage irregularities arising from local sequence architecture, and were largely avoidable, either by improving enrichment efficiency or increased coverage, as shown by the results obtained with the Illumina NGS-LSD assay. Filter 2 was also adjusted by checking variants by Sanger sequencing (see next section) and comparing data from two different variant-calling software packages, Lifescope and GATK. Variants obtained from HiSeq2000 passed through the same Filter 1 as in SOLiD4, reducing the number of variants directly to 68. In this case, a second filter was not necessary as the high coverage achieved meant that the false-positive rate was insignificant using HiSeq2000.

### Assessment of the novel genetic tool for LSD diagnosis

All putatively pathogenic variants detected by either of the two platforms were subsequently tested by Sanger sequencing of both the index case and parents, and all were confirmed. A more thorough analysis of the variants detected by SOLiD4 was carried out, to test the reliability of Filter 2 in discrimination of real from false variants. Of the 71 missense variants which passed Filter 2 and were confirmed with Sanger sequencing (see previous section), 83% were found to be real and 17% false-positive. Of 48 variants which did not pass through Filter 2, 14 were additionally selected for having values near the limit for one or more conditions; all of these were then verified as false-positive. Therefore, while overall we had a false-positive rate of 17%, we conclude that the second filter detected real variants with a high degree of confidence. If, on the other hand, we had only allowed variants fulfilling all 4 conditions of Filter 2 to pass through, we would not have had any false positives but instead we would have had a rate of 9% of false negatives. A comparison of the two variant calling software packages was also found to be useful in discriminating between real and false variants, as none of the false positives seen using Lifescope appeared when using GATK and vice versa.

### Detection of gross deletions

**Figure 2:**
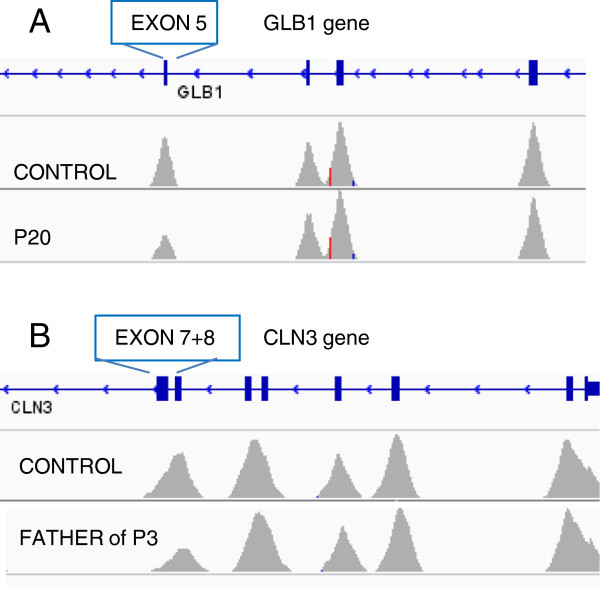
**Heterozygous macrodeletions detected by LSD-NGS. A)** GLB1 exon 5 deletion; **B)** CLN3 exon 7 + 8 deletion. The area of the coverage peak for each deleted exon is half that seen in the corresponding control sample.

One advantage of NGS-based strategies, as opposed to Sanger sequencing, is that, in addition to SNVs and small indels, they can also detect gross deletions and insertions which affect one or more exons. Thus, we detected two heterozygous macrodeletions, one an exon 5 deletion in *GLB1* (P20), and another an exon 7 + 8 deletion in *CLN3* (C18) (Figure 2). We also detected the same *CLN3* macrodeletion in a homozygous state (C2 and P3; not shown).

### Limitations of SNP calling software

Using Bioscope software, heterozygous variants were found with a percentage of NRA reads between 30 and 50%, while homozygous variants had percentages of between 70 and 100%. Lower percentages were seen for small deletions and insertions, comprising a potential problem with this technology. On the other hand, a comparison of the two SNP calling software packages showed there to be a significant proportion of false negatives with each package. Thus, of 105 variants confirmed by Sanger sequencing, Bioscope had failed to detect 3 (2.8%) and GATK failed to detect 9 (8.5%). This illustrates the importance of using more than one type of variant calling software in parallel, to discriminate between real and false variants as well as to reduce the false-negative rate as much as possible.

### Diagnosis achieved with the NGS-LSD screening tool

**Figure 3:**
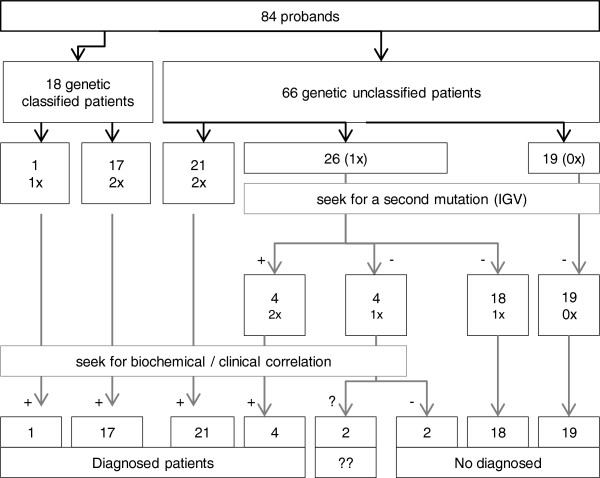
**Diagnosis achieved using NGS-LSD.** (1x: one mutation found; 2x: two mutations found in the same gene; VCS: variant calling software; ??: not confirmed.)

We applied the NGS-LSD method to 84 probands with a spectrum of early-onset neurodegenerative disorders that were potentially caused by a deficiency in one of the 57 LSD proteins (Figure 3). From these 84, we selected 18 control samples with mutations previously identified by Sanger sequencing, three of which had only a single mutation in the affected gene (C14, 15, 18). Of the 33 mutations expected, all but one was detected using the NGS-LSD assay (Table 2). The undetected mutant was located on exon 1 of the *NAGLU* gene (C8), which was not covered by hybridization baits, due to its being located close to repetitive regions. It is important to highlight that this mutation would have been detected with Illumina platform. For the 66 unclassified patients, an LSD suspicion index was assigned, as low, moderate, or high in each case. For each case, clinical data and tests carried out for diagnostic purposes, including genetic, were collected (Table 3). Based on the presence of one or two mutations, we were able to achieve genetic diagnosis in 26 patients. Twenty-two of these were found to carry two mutations in the same gene, consistent with clinical and/or biochemical features and establishing a genetic diagnosis (Table 4). A further 26 patients were found to be carriers of mutations (Tables 4 and 5). In all cases, the gene harboring a mutation was carefully examined to find any other variant that might have been initially undetected due to its location in an intronic region, synonymity, or being a gross indel. Thus, a second mutation was found in 4 patients (P11, 20, 21, 23). Of the remaining carriers, we carried out biochemical assays, to confirm or exclude disease, in four patients (P12, 27, 28, 29) chosen for the severity of the variant found and correlation between genotype and phenotype. Biochemical tests on P27, P28 and P29 gave negative results. In P12 the diagnosis of NPC2 could not be confirmed but it could not be discarded neither (filipin staining was unconclusive but this is not unusual in a percentage of NPC2 patients and only one heterozygous pathogenic mutation was found). It is important to emphasize that in the current study we were readily able to confirm biochemically mutation pathogenicity in the majority of genes analyzed, but this is not possible for pathologies that have no biochemical markers and in which confirmation will need to be obtained by other methods. No mutations were found in 19 probands (p49-p65), but the LSD index of suspicion was low for all but one of these.

**Table 2:** Results obtained for positive controls included in NGS-LSD assay

**CC**	**GENE**	**REF_SEC**	**NT CHANGE**	**AA CHANGE**	**ZIG**	**MD**	**DIAGNOSIS**	**OMIM**
C1	ARSA	NM_000487	c.1046delC	p.P349fs	HO	+	Metachromatic leukodystrophy	250100
C2	CLN3	NM_000086	c.461-280_677 + 382del966	p.[Gly154Alafs*29, Val155_Gly264del]	HO	+	Ceroid lipofuscinosis, neuronal, 3	204200
C3	FUCA1	NM_000147	c.464C > T	p.S155F	HT	-	Fucosidosis	230000
	FUCA1	NM_000147	c.790C > T	p.R264X	HT	-		
C4	GALNS	NM_000512	c.281G > T	p.R94L	HO	+	Mucopolysaccharidosis type IVA	253000
C5	GLB1	NM_000404	c.1581G > A	p.W527X	HO	+	GM1 gangliosidosis	230500
C6	GNPTAB	NM_024312	c.1208 T > C	p.I403T	HT	+	Mucolipidosis III alpha/beta	252600
	GNPTAB	NM_024312	c.1999G > T	p.E667X	HT	+		
C7	GUSB	NM_000181	c.526C > T	p.L176F	HT	+	Mucopolysaccharidosis VII	253220
	GUSB	NM_000181	c.530C > T	p.T177I	HT	-		
C8	NAGLU	NM_000263	c.900C > T	p.R234C	HT	+	Sanfilippo B	252920
C9	NEU1	NM_000434	c.700G > A	p.D234N	HT	+	Sialidosis	256550
	NEU1	NM_000434	c.1021C > T	p.R341X	HT	+		
C10	SGSH	NM_000199	c.120C > G	p.Y40X	HO	+	Sanfilippo A	252900
C11	SMPD1	NM_000543	c.739G > A	p.G247S	HO	+	Niemann-Pick disease, type A	257200
C12	TPP1	NM_000391	c. 622C > T	p.R208X	HO	+	Ceroid lipofuscinosis, neuronal, 1	256730
C13	CTSA	NM_000308	c.448G > A	p.V150M	HT	-	Galactosialidosis	256540
	CTSA	NM_000308	c.284delC	p.P95Lfs	HT	-		
C14	NPC1	NM_000271	c.1552C > T	p.R518W	HT	+	Niemann-Pick disease, type C	257220
C15	NPC1	NM_000271	c.2594C > T	p.S865L	HT	+	Niemann-Pick disease, type C	257220
C16	SLC17A5	NM_012434	c.918 T > G	p.Y306X	HT	+	Sialic acid storage disorder,infantile	269920
	SLC17A5	NM_012434	c.500 T > C	p.L167P	HT	-		
C17	ARSB	NM_000046	c.427delG	p.V143Sfs	HO	+	Mucopolysaccharidosis VI	253200
C18	CLN3	NM_000086	c.461-280_677 + 382del966	p.[Gly154Alafs*29, Val155_Gly264del]	HT	+	Ceroid lipofuscinosis, neuronal, 3	204200

CC: Control Code (C1-C12 processed with SOLiD4 platform; C13-C18 processed with HiSeq2000); REF SEQ: reference sequence for which mutations are annotated; NT and AA CHANGE: nucleotide and amino acid change; ZIG: zigosity; HO: homozygotes; HT: heterozygotes; MD: Mutation description in Human Gene Mutation Database (+: previously associated with pathology; −: not previously associated).

**Table 3:** Diagnostic suspicions and biochemical/histopathological tests for patients diagnosed using the NGS-LSD tool

**PC**	**AO**	**AD**	**GD**	**NGS-LSD**	**Initial signs**	**Diagnostic suspicions**	Biochemical-histopathology studies*	Other genetic studies*
P1	2Y	-	12Y	CLN5	Clumsiness, frequent falls, apraxic gait	CLN	VL; SBIOP + .	PPT1, TTP1
P2	4Y	-	7Y	CLN6	Clumsiness, myoclonic movements	GM2, GCL, MLD, Fabry, Schindler, MANSA, MANBA, MPS, CLN	EA: HEXA, HEXB, GLA, NAGA, MANBA, MAN2B1, MPS, GALC, ARSA, PPT1, TTP1	MFSD8
P3	3Y	-	16Y	CLN3	Language regression, school inattention, social isolation	CDG, GM1, GM2, GSL, Schindler, GSD II, III, CLN	VL; FC; SPA ; EA: GLB1, HEXB, CTSA, NAGA, GAA, AGL; SBIOP + .	
P4	2Y	-	23Y	CLN7	Language delay, clumsiness	CLN	SBIOP +; EA: CLN2.	CLN1
P5	4Y	-	16Y	CLN3	Conduct disorder, attention deficit disorder	CLN	VL +; SBIOP, MBIOP, NBIOP + .	PPT1, TTP1, CLN3, CLN5, CLN8
P6	3Y	4Y	7Y	MLD	Leukodystrophy	Spastic paraparesia	EA: ARSA +	
P7	1Y	-	9Y	GM1	Global developmental delay, gait instability	CDG; RCCD; INAD	MBI; NBIOP +; MBS; SPA; EA: HEXA, HEXB	
P8	2Y	7Y	4Y	GM2	Language delay, conduct disorder	Unspecific global developmental delay, Neurodegenerative disease; CLN, GM2	SBIOP; MBS; EA: HEXA + .	Caryotype; Fragile X; Smith-Magenis
P9	2Y	-	14Y	GM2	Clumsiness, frequent falls	Ataxia, Attention deficit disorder	MBI	Caryotype; Fragile X; CGH-60 k array.
P10	3Y	9Y	12Y	GM1	Clumsiness, frequent falls, language delay	RS, MPSIII, GM1	MBS, U-GAGs; EA: GLB1+	Caryotype, Fragile-X, MECP2
P11	5Y	-	22Y	GM2	Learning problems, language difficulties	CLN	SBIOP	CLN3, CLN8
P12	13Y	-	18Y	NPC2^#^	Clumsiness, short stature	MTDPS; RCCD; SCA; DRPLA	MBIOP; RCCFS; EA: muscular Coenzima Q10	mtADN(muscle); MTATP6; tRNALeu(UUR); SCA1,2,3,6,7,12,17; ATN1
P13	18 M	-	4Y	ML II,III	Growth delay, macrocephaly	MPS	U-GAGs	SHOX
P14	12 M	3Y	3Y	GM2	Psycomotor regression, language regression	RS, GM2	EA: HEXA +, HEXB	MECP2
P15	0 M	3 M	1Y	GM1	Hypotonia, hepatomegaly, cardiomyopathy, bony abnormalities	GSD II	EA: GAA	
P16	9 M	2Y	2Y	MPSVI	Bilateral costal deformity, macrocephaly	AA, MPS	U-GAGs, Dermatan sulfate in urine, EA: ARSB	
P17	0 M	-	2Y	ML II,III	Hypotonia, microcephaly, jaundice	MPS	MBS, U-OLG, U-GAGs	Caryotype; CGH array
P18	2 M	3Y	6Y	MPSII	Bilateral inguinal hernia	MPS	U-GAGs; EA: IDS +	
P19	1Y	4Y	5Y	MPSI	Clubfoot, arthrogryposis, clawed hands	MPS	U-GAGs; EA: IDUA +, IDS, ARSB	
P20	0 M	1Y	1Y	GM1	Hypotonia, severe psycomotor delay	GM1, GM2, GSL, ML I	MBS; VL; U-GAGs; EA: GBL1 +, HEXA, HEXB, CTSA, NEU1	
P21	18 M	3Y	3Y	GM2	Unsteady gait, frequent falls	TORCH, Leukodystrophy	EA:	
P22	2Y	-	6Y	CLN8	Cognitive regression, epilepsy	Lennox syndrome, CLN	PBIOP +	
P23	3Y	-	9Y	CLN10	Psycomotor regression, dystonia, epilepsy	Neuroaxonal dystrophy, sphingolipidosis	MBIO	mtDNA
P24	3Y	5Y	23Y	MPSIIIB	Language delay, conduct disorder, learning disabilities, hypoacusia	MPS	Heparan sulfate +	
P25	1Y	-	6Y	GM2AB	Developmental delay, regression	GM2, GSD II	EA: HEXA, HEXB, GAA (+); HPS GM2 +	GAA
P26	4Y	-	25Y^†^	CLN8	Cognitive regression, epilepsy	CLN		CLN6

*Abbreviations:PC* Patient Code, *AO* Age of Onset (Y: years; M: months; ^†^deceased), *AD* Age at Diagnosis, *GD* age at Genetic Diagnosis, *NGS-LSD* diagnosis reached with our NGS tool, *CLN* Neuronal Ceroid Lipofuscinosis, *MLD* metachromatic leukodystrophy, *GM1 and 2* Gangliosidosis I and II, *NPC2* Niemann-Pick disease, type C2, *ML II III* mucolipidosis III alpha/beta, *MPS* mucopolysaccharidosis, *GM2AB* Gangliosidosis type II, AB variant, *MANSA* Mannosidosis, alpha-, types I and II, *MANBA* mannosidosis beta, *CDG* congenital disorder of glycosylation, *GSL* galactosialidosis, *GSD* glycogen storage disease, *GSD II* Pompe disease, *RCCD* respiratory chain complex deficiency, *INAD* infantile neuroaxonal dystrophy, *RS* Rett syndrome, *AA* autoimmune arthritis, *ML I* sialidosis, *TORCH* Band-like calcification with simplified gyration and polymicrogyria, *MBS* metabolic study, *SPA* sialotransferrin profile analysis, *VL* vacuolated lymphocytes in peripheral blood, *FC* foam cells in bone marrow, *RCCFS* respiratory chain complex functional studies, *GCL* globoid cell leukodystrophy or Krabbe disease, *SCA* spinocerebellar ataxia, *EA* enzymatic assay, *U-GAGs* urinary glycosaminoglycan levels, *U-OLG* urinary oligosaccharides levels, *SBIOP* skin biopsy, *MBIOP* muscle biopsy, *NBIOP* nerve biopsy, *HPS* pathological study, *mtDNA* mithocondrial DNA deletions, depletion and punctual mutations, *MTDPS* mitochondrial depletion syndrome, *CGH* comparative genomic hybridization; ^#^: not confirmed; *All results were negative unless indicated by a plus sign.

**Table 4:** Diagnosis achieved using the NGS-LSD tool

**PC**	**AO**	**GDD**	**IS**	**GENE**	**OMIM**	**REF SEC**	**NT CHANGE**	**AA CH**	**MT**	**SS**	**PP**	**CS**	**ZIG**	**MD**	**BA**	**DIAGNOS**
P1	2Y	10Y	Mod	CLN5	608102	NM_006493	c.335G > C/c.835G > A	p.R112P/p.D279N	099/0.99	0/0.16	1/1	+	HO	+	NA	Finnish variant late infantile CLN
P2	4Y	4Y	Mod	CLN6	606725	NM_017882	c.794_796del	p.S265del	-	-	-		HO	+	NA	Early juvenile late infantile CLN
P3	3Y	12Y	High	CLN3	607042	NM_000086	c.461-280_677 + 382del966	p.[Gly154Alafs*29, Val155_Gly264del]	-	-	-		HO	+	NA	Juvenile CLN
P4	2Y	21Y	Mod	MFSD8	611124	NM_152778	c.881C > A	p. T294K	0.99	0	0.99	+	HO	+	NA	Turkish variant late infantile CLN
P5	4Y	12Y	High	CLN3	607042	NM_000086	c.371_372insT	p.Y124fs	-	-	-		HO	-	NA	Juvenile CLN
P6	3Y	4.5Y	High	ARSA	607574	NM_000487	c.465 + 1G > A	----------	-	-	-	+	HO	+	+	Metachromatic leukodystrophy
P7	1Y	8Y	Low	GLB1	611458	NM_000404	c.922 T > C	p.F308L	0.99	0	1	+	HO	-	+	GM1 gangliosidosis
P8	3Y	1Y	High	HEXA	606869	NM_000520	c.533G > A	p.R178H	0.99	0	1	+	HO	+	+	GM2 gangliosidosis, B1variant
P9	4Y	10Y	Low	HEXA	606869	NM_000520	c.1496G > A	p.R499H	0.99	0	1		HT	+	+	GM2 gangliosidosis, juvenil (TS)
				HEXA		NM_000520	c.1003A > T	p.I335F	0.99	0	0.97		HT	+		
P10	3Y	7Y	High	GLB1	611458	NM_000404	c.602G > A	p.R201H	0.99	0.02	1		HT	+	+	GM1 gangliosidosis
				GLB1		NM_000404	c.1188_1188dupG	p.P397fs	-	-	-		HT	-		
P11	5Y	18Y	High	HEXA	606869	NM_000520	c.155C > A	p.S52X	-	-	-	+	HT	+	+	GM2 gangliosidosis juvenile (TS)
				HEXA		NM_000520	c.1305C > T	p.Y435Y	-	-	-		HT	-		
P12	13Y	6Y	Low	NPC2		NM_006432	c.441 + 1G > A	-----------	-	-	-		HT	-	-	Niemann-Pick disease, type C2?
P13	18 M	3Y	High	GNPTAB	607840	NM_024312	c.2354 T > G	p.L785W	0.71	0.09	0.022	+	HT	-	+	Mucolipidosis II/III
				GNPTAB		NM_024312	c.1774G > A	p.A592T	0.98	0.01	0.955		HT	-		
P14	12 M	4Y	High	HEXA	606869	NM_000520	c.718_719insT	p.K240fs					HT	-	+	GM2 gangliosidosis (TS)
				HEXA		NM_000520	c.1003A > T	p.I335F	0.99	0.00	0.467		HT	+		
P15	0 M	1Y	High	GLB1	611458	NM_000404	c.671_672delAT	p.H224Qfs				+	HO	-	+*	GM1 gangliosidosis
P16	9 M	1Y	High	ARSB	611542	NM_000046	c.382_384delCTC	p.L128del					HO	-	+*	Mucopolysaccharidosis VI
P17	0 M	3Y	High	GNPTAB	607840	NM_024312	c.3739_3742delCTTT	p.E1248fs				+	HO	-	+	Mucolipidosis II/III
P18	2 M	6Y	High	IDS	300823	NM_000202	c.425C > T	p.S142F	0.98	0.00	0.998	+	HE	+	+*	Hunter Syndrome
P19	3Y	5Y	High	IDUA	252800	NM_000203	c.1205G > A	p.W402X				+	HT	+	+*	Hurler Syndrome
				IDUA		NM_000203	c.1874A > G	p.Y625C	0.98	0.00	0.99		HT	-		
P20	10 M	2Y	High	GLB1	611458	NM_000404	c.947A > G	p.Y316C	0.999	0	0.79		HT	+	+*	GM1 gangliosidosis
				GLB1		NM_000404	c.458-401_552 + 1033del1529	-----------					HT	+		
P21	20 M	4Y	High	HEXA	606869	NM_000520	c.459 + 5G > A	-----------					HT	+	+*	GM2 gangliosidosis (TS)
				HEXA		NM_000520	c.533G > A	p.R178H	0.99	0	1		HT	+		
P22	2Y	4Y	High	CLN8	607837	NM_018941	c.509C > G	p.T170R	0.999	0.00	0.999	+	HO	+	NA	Ceroid lipofuscinosis, neuronal, 8
P23	3Y	9Y	High	CTSD	116840	NM_001909	c.470C > T	p.S157L	0.98	0.03	0.005	+	HT	-	NA	Ceroid lipofuscinosis, neuronal, 10
				CTSD		NM_001909	c.353-12G > A	-----------	-	-	-		HT	-	pend^ε^	
P24	3Y	23Y	High	HGSNAT	610453	NM_152419	c.1250 + 1G > A	-----------	-	-	-		HT	+	+*	Sanfilippo C
				HGSNAT		NM_152419	c.1270G > A	p. G424S	0.999	0.59	1		HT	+		
P25	1Y	6Y	High	GM2A	613109	NM_000405	c.333delC	p. C112Vfs	-	-	-		HO	-	+^#^	GM2 gangliosidosis, AB variant
P26	4Y	>20Y	High	CLN8	607837	NM_018941	c.792C > G	p. N264K	0.97	0	0.99	+	HO	-	NA	Ceroid lipofuscinosis, neuronal, 8

Notes and abbreviations: Samples from Patients P1-P12 were processed using SOLiD4 (Life Technologies), but those from P13-P26 using HiSeq2000 (Illumina); PC: Patient Code; AO: Age of Onset (Y: years; M: months); GDD: Genetic Diagnosis Delay (Y: years; M: months: DEC: deceased); IS: index of suspicion of LSD (Mod: moderate); REF SEC: reference sequence for which mutations are annotated; Nt and Aa Change: nucleotide and amino acid change (population frequency ≤0.01 according to 1000G2012 database); MT: Mutation Taster, SS: Sift Score and PP: Polyphen 2 score (in silico values to assess pathogenicity of missense variants); CS: Cosegregation; ZIG: zigosity; HO: homozygote; HT: heterozygote; HE: hemizygous; MD: Mutation description in Human Gene Mutation Database (+: previously associated with pathology; −: not previously associated); BA: Biochemical assay (+: positive; NA: test not available; −: negative); CLN: Neuronal Ceroid Lipofuscinosis; TS: Tay-Sachs; *Levels detected before NGS-LSD assay; ^ε^mRNA splicing analysis; ^#^histopathology congruent with GM2 diagnosis.

**Table 5:** Results found for patients remaining un-diagnosed

**PC**	**IS**	**Gene**	**Ref sec**	Coding variants*		**dbSNP ID**	**MAF**	**Zig**	**MD**	**BA**
P27	High	MANBA	NM_005908	c.1922G > A	p.R641H	--		HT	+	Neg^Ψ^
P28	High	HYAL1	NM_033159	c.676C > T	p.R226C	--		HT	-	NCI
		HEXB	NM_000521	c.383 T > G	p.L128R	--		HT	-	Neg^Ψ^
P29	High	SMPD1	NM_000543	c.1460C > T	p.A487V	--		HT	+	Neg^Ψ^
P30	Low	NEU1	NM_000434	c.1070G > A	p.R357Q	--		HT	-	NCI
P31	Mod	MFSD8	NM_152778	c.50C > G	p.T17R	--		HT	-	NCI
P32	Low	NAGA	NM_000262	c.697G > A	p.V233M	--		HT	-	NCI
		NPC1	NM_000271	c.665A > G	p.N222S	rs1805081	0.001	HT	+	NCI
P33	Low	SMPD1	NM_000543	c.1550A > T	p.E517V	--		HT	+	NCI
P34	High	SGSH	NM_000199	c.308A > G	p.K103R	--		HT	-	NCI
		CLN6	NM_017882	c.755G > C	p.R252P	--		HT	-	NCI
P35	Low	TPP1	NM_000391	c.1117C > G	p.Q373E	--		HT	-	NCI
		GAA	NM_000152	c.1367G > T	p.R456M	--		HT	-	NCI
P36	High	CLN5	NM_006493	c.606G > A	p.M202I	--		HT	-	NCI
P37	High	SMPD1	NM_000543	c.8G > A	p.R3H	--		HT	-	NCI
P38	Low	IDUA	NM_000203	c.251G > C	p.G84A	--		HT	-	NCI
P39	Low	IDS	NM_000202	c.754G > A	p.D252N	--		HO	+	Neg*
P40	Low	CLN3	NM_000086	c.995C > T	p.A332V	--		HT	-	NA
P41	High	CLN5	NM_006493	c.726C > A	p.N242K	--		HT	-	NA
P42	High	IDUA	NM_000203	c.650G > A	p.R217Q	--		HT	-	NCI
		NPC1	NM_000271	c.2257G > A	p.V753M	--		HT	-	NCI
P43	Mod	ASAH1	NM_177924	c.2 T > C	p.M1T	--		HT	-	NCI
P44	Low	SMPD1	NM_000543	c.1460C > T	p.A487V	--		HT	+	NCI
P45	Low	CLN3	NM_000086	c.995C > T	p.A332V	--		HT	-	NA
P46	Low	CLN5	NM_006493	c.606G > A	p.M202I	--		HT	-	NA
		GALNS	NM_000512	c.1127G > A	p.R376Q	--		HT	+	NCI
P47	Low	GNPTG	NM_032520	c.857C > T	p.T286M	--		HT	+	NCI
		NPC1	NM_000271	c.3535A > G	p.M1179V	rs61731969	0.002	HT	-	NCI
P48	Low	MAN2B1	NM_000528	c.844C > T	p.P282S	rs45576136	0.003	HT	-	NCI

Notes and abbreviations: Samples from P27-39 were processed using SOLiD4; those from P40-P48 using HiSeq2000; P49-P66 no mutation was found. PC: Patient Code; IS: Index of LSD Suspicion (Mod: moderate); *detected with frequency ≤0.01 in 1000 g2012 database; MAF: minor allele frequency; Zig: zygosity; HO: homozygote; HT: heterozygote; MD: Mutation description in Human Gene Mutation Database (+: previously associated with pathology; −: not previously associated); BA: Biochemical assay (+: positive; NA: test not available; Neg: negative; NCI: not clinically indicated; Ψ Enzimatic assay; *GAGs).

### Unexpected diagnoses

In cases P7, P9 and P11, the diagnoses were unexpected, adding significant value to our method. In case P7, the patient’s history did not initially suggest type 1 gangliosidosis (GM1), due to presence of cerebellar atrophy on magnetic resonance imaging (MRI) at first consultation (age 2 years) and a consistently raised lactic acid level in both blood and cerebrospinal fluid (CSF). Together with updated clinical data, therefore, a mitochondrial cytopathy seemed likely and a muscle biopsy was performed, which showed a slight deficiency in complex I of the mitochondrial respiratory chain. Subsequent electroneurography (at 4.7 years) showed results consistent with a mixed polyneuropathy. This finding, along with the clinical data, MRI, and minimal deficiency in complex I, suggests a probable neuroaxonal dystrophy. With enzymatic and genetic data from the NGS-LSD assay, we concluded that this child suffered from a late infantile GM1 that could be categorized as atypical due to the presence of cerebellar atrophy, mixed polyneuropathy, and increased lactate in the blood and CSF. In classical GM1, MRI is usually normal or shows some late brain atrophy, while polyneuropathy and high lactate levels are not usually present.

Case P9 was shown to have juvenile type 2 gangliosidosis (GM2), following a delayed suspicion of a storage disorder due to the late onset and low specificity of symptoms, and slow progression of the disease. In case P11, ceroid lipofuscinosis neuronal (CLN) was initially suspected due to epilepsy, cognitive regression, and a loss of memory and expressive language. Progressive multifocal epilepsy was refractory to combination therapy, the patient’s vision worsened, and he appeared ataxic and lost bowel control. There was also a reduced retinal vascular tree with papillary pallor, thought to signify the onset of retinopathy; however the detection of a severe mutation in the GM2-associated gene *HEXA* altered the diagnosis. P11 currently has progressive optic atrophy. Whilst infantile GM2 is easy to recognize due to a cherry-red spot in the fundus, juvenile GM2 is difficult without the identification of genetic variants, as discussed above. As it is shown in the Table 4, second mutation in *HEXA* for P11 was synonymous. Familial co segregation was demonstrated and the program Human splicing finder predicted the disruption of an enhancer motif for SRp40 protein and the simultaneous creation of a new silencer motif.

A finding of interest was the presence of a hemizygous variant of the Hunter-associated gene *IDS* in patient P25. While this mutation is already registered in Human Gene Mutation Database (HGMD) as associated with the Hunter phenotype, the patient showed none of the clinical features usually found in Hunter patients; he did not showed increased levels of urine GAGs and no skeletal deformities were shown in radiological test, excluding completely a Hunter diagnosis. This lends further weight to what has long been suspected, that we have only seen the ‘tip of the iceberg’ of genotype-phenotype associations in clinical genetics and that NGS will change some universally accepted paradigms and lead to new genetic prescripts, such as the implication of more than one gene in classically monogenic disorders [32].

### Time-to-diagnosis and cost: comparisons with Sanger sequencing

The period between onset of disease and genetic diagnosis (GDD column, Table 4) also enhances the value of the NGS-LSD tool. Diagnostic delay in our study was on average 7 (range 1–23) years. Using NGS-LSD ended 14–15 diagnostic odysseys, thus opening the way for genetic counseling and carrier tests. In terms of cost, the difference between classical and next-generation sequencing technology is $2400 and $0.1 per million bases, respectively. Two examples from the current study serve to illustrate the difference between two technologies. Although a CLN was strongly suspected, patient P5 (with 13 years of diagnostic delay) underwent five classical genetic analyses without a positive result and at a total cost of about 4000€. With an early NGS-LSD assay, the diagnosis could have been made in 8 weeks at a cost of about 900€. In case P3 several diseases were suspected over a period of 12 years, including GM1, Sandhoff, galactosialidosis, Schindler disease and CLN. If the NGS-LSD tool had been used on first suspicion, the time-to-diagnosis would have been reduced to 1–2 months, and the cost to that of a single genetic analysis.

### Future approaches to reduce non-diagnosis

The current study ended with 39–40 patients still undiagnosed, although 64% of these had a low or moderate index of suspicion of LSD. Clearly, these undiagnosed cases indicate a significant problem with our proposed tool, which is due to the considerable phenotypic overlap between many LSD and non-LSD neurodevelopmental conditions for which the genetic variants are not included in the NGS-LSD technique. To address this problem, we are currently developing a broad-range genetic panel that encompasses most known neurometabolic disorders, the NGS-NMD1 tool, which will be updated as new genes come to light with continued research. Such an approach will form an important next step in optimizing the neurometabolic diagnostic process. Six of our undiagnosed patients are currently undergoing whole-exome resequencing.

### Application of NGS-LSD to clinical diagnosis

NGS technologies have significantly improved sequencing capacity in the past 5 years. These technologies are now widely used for research purposes and are starting to find their way into clinical applications [33-35]. Whole-genome and exome sequencing approaches are being successfully implemented in research projects [31,36-42], but are not yet routine strategies in diagnosis due to high costs, long turnover times (run and analysis time) and ethical issues. Targeted resequencing, on the other hand, is appealing in a clinical setting due to lower sequencing costs, shorter sequencing time, simpler data analysis, and greater sensitivity per gene due to the greater coverage achieved [43,44]. The results of the current study also illustrate the importance of good coverage to reliability in detection of mutations in NGS in clinical settings.

Due to the nature of the technology, NGS also brings with it uncertainties and limitations of a higher order of magnitude than previously seen in genetic diagnosis. However, uncertainty in genetic testing is nothing new, and we also have the foresight and fortitude to develop tools to deal with these issues. Assays must also be carefully validated with pilot projects to assess sensibility and accuracy of these new technologies. Several groups are working in this direction, designing resequencing assays for a panel of genes related to groups of diseases that have similar clinical manifestations and difficult diagnoses [45-54].

To the best of our knowledge, the current study is the first to use NGS in LSD screening. Overall, it shows that a high rate of detection of mutations is possible when sequence coverage is sufficient and gaps due to limitations in the enrichment method can be overcome. Our results show that the combination of in-solution based capture and NGS can be used for the parallel screening of multiple disease genes and can successfully identify disease-causing mutations. This assay, therefore, can be used as a support genetic tool, that always in combination with biochemical and clinical data could facilitate a diagnosis. Finally, we have shown the power of our approach as a tool for making diagnoses that are particularly challenging and for bringing diagnostic odysseys to a more rapid conclusion. It will be important, therefore, to continue to work with specialists to optimize this powerful and promising technology for the benefit of patients and their families”.

## Abbreviations

LSDs: Lysosomal storage disorders; NGS: Next-generation sequencing; ERT: Enzyme-replacement therapy; MPS: Mucopolysaccharidoses; SETS: SOLiD experimental Tracking Software; GATK: Genome Analysis Toolkit; RTA: Illumina Real Time Analysis; GEM: Genome Multitool; BFAST: BLAT-like Fast Accurate Search Tool; SNVs: Single nucleotide variants; LSMDs: Locus Specific Mutation Databases; SIFT: Sorting Intolerant From Tolerant; NRA: Non-reference allele; MQV: Mean quality values; GM1: Type 1 gangliosidosis; GM2: Type 2 gangliosidosis; MRI: Magnetic resonance imaging; CSF: Cerebrospinal fluid; CLN: Ceroid lipofuscinosis neuronal; HGMD: Human gene mutation database.

## Competing interests

The authors declared that they have no competing interest.

## Authors’ contributions

AFM was involved in the study design, choice of genes, interpreting the NGS data, DNA sample and clinical data collection and writing the manuscript. MM collaborated in study design, choice of genes, DNA extraction, in solution enrichment and sequencing in SOLID platform, and Sanger sequencing of detected variants. JE, MP, MCG, MLC, JMF, MSPP and JA collaborated in the patients and clinical data collection, evaluation of data analysis results and managing biochemical and biopsy test to support results. JMF also helped to write manuscript. LL collaborated with blinded controls and some patients. SG was involved in DNA extraction, sample preparation for NGS assays, Sanger sequencing of detected variants and draft manuscript correction. DC and JAC coordinated and integrated the different works, especially in the laboratory landscape and helped to elaborate final manuscript. All authors read and approved the final manuscript.
